# Neutrophil autophagy induced by monosodium urate crystals facilitates neutrophil extracellular traps formation and inflammation remission in gouty arthritis

**DOI:** 10.3389/fendo.2023.1071630

**Published:** 2023-09-22

**Authors:** Shanshan Huang, Yaohui Wang, Shibo Lin, Wei Guan, Hui Liang, Jiajia Shen

**Affiliations:** ^1^ Department of Endocrinology, The Affiliated Jinling Hospital of Nanjing University Medical School, Nanjing, China; ^2^ Department of Pathology, Jiangsu Province Hospital of Chinese Medicine, Affiliated Hospital of Nanjing University of Chinese Medicine, Nanjing, China; ^3^ Department of General Surgery, First Affiliated Hospital, Nanjing Medical University, Nanjing, China

**Keywords:** gouty arthritis, neutrophil extracellular traps, autophagy, ATG7, p53, inflammation remission

## Abstract

Neutrophil extracellular traps (NETs) are composed of chromatin filaments coated with granular and cytosolic proteins, which contribute to the pathogenesis and progression of immune-related diseases. NETs are frequently observed in gouty arthritis, but the related mechanisms remain poorly understood. The aim of our study was to systematically elucidate the molecular mechanisms of self-remitting effects in gouty arthritis, and the causative relationship between neutrophil autophagy and NETs. The air pouch and paw edema model were used to simulate gouty arthritis in mice. Neutrophil infiltration and the formation of NETs were found in gouty arthritis. Interestingly, monosodium urate (MSU) crystals could induce the formation of NETs, degrade inflammatory factors, and alleviate the inflammatory response in gouty arthritis. In addition, MSU crystals resulted in profound molecular alterations in neutrophils using RNA-seq analysis, including autophagy activation. MSU crystals could activate neutrophil autophagy *in vitro*, and autophagy activators and inhibitors could regulate the formation of NETs. Furthermore, we explored the mechanism of autophagy-induced NETs. Autophagy related protein 7 (ATG7) produced by neutrophils stimulated with MSU crystals worked synergistically with p53 to enter the nucleus, promoting peptidyl arginine deiminase 4 (PAD4) expression, and inducing the formation of NETs. Finally, we substantiated that neutrophil autophagy regulates the severity of gouty arthritis *via* the formation of NETs in PAD4 ^-/-^ mice. Our results indicated that the autophagy of neutrophils regulates the formation of NETs and degrades inflammatory factors. Regulating autophagy and interfering with the formation of NETs represents a potential therapeutic approach against gouty arthritis during clinical practice.

## Introduction

Gouty arthritis is self-limited aseptic arthritis caused by decreased purine metabolism disorder and uric acid excretion (1, 2). It is widely acknowledged as the most common inflammatory arthritis, with a worldwide prevalence ranging from below 1% to 6.8% (3). The morbidity among the Chinese population has been increasing gradually in recent years. From 2000 to 2014, the prevalence of hyperuricemia and gout in mainland China was 13.3% and 1.1%, respectively (4). The self-limiting nature of the disease is one of the significant characteristics distinguishing gouty arthritis from other joint diseases or autoimmune diseases (5). Therefore, effective treatment strategies based on self-limitation mechanisms in gout have been increasingly emphasized.

It is well-recognized that gouty arthritis results from the inflammatory response to monosodium urate (MSU) crystals (6, 7). The core event in inflammation caused by MSU crystals remains the activation of leukocytes, which leads to the initiation of the inflammatory cascade (8). Neutrophils are recruited to the inflammatory site and release cytokines and mediators, such as IL-1β, IL-6, TNF-α, NLRP3 inflammasome, ROS, metalloproteinases, and various lysosomal enzymes, to amplify inflammatory reactions in the joints (9, 10). After amplification of the inflammatory cascade, patients undergoing an acute attack experience relief within a few days. Cumulative evidence suggests that mechanisms associated with the improvement of gouty arthritis involve regulators of pro-inflammatory cytokines, neutrophils, negative regulators of inflammasome and TLR signaling (11, 12). Neutrophil-derived microvesicles mediated control of inflammasome-driven inflammation represents a promising concept of autoregulation of neutrophil activation in gout (13).

Furthermore, recent research indicates that increased autophagy activity also plays an important role in relieving gouty arthritis (14). Natural agonists of autophagy have also shown anti-inflammatory effects in gouty arthritis (15). After stimulation of neutrophils with 2-Acetoxy-1-methoxypropane (PMA), intracellular chromatin depolymerization in response to ROS and cellular autophagy led to the formation of NETs. Inhibition of neutrophil autophagy with 3-methyladenine did not affect the NADPH-dependent ROS burst, but inhibited intracellular chromatin depolymerization and subsequent NETosis, ultimately led to apoptosis (16). However, the underlying mechanisms remain poorly understood, warranting further studies.

The present study documented that NETs induced by MSU crystals degraded inflammatory factors and alleviated the inflammatory response in gouty arthritis. Furthermore, MSU crystals could stimulate neutrophil autophagy. In addition, we further revealed that Autophagy related protein 7 (ATG7) upregulation in neutrophils promoted the expression of peptidyl arginine deiminase 4 (PAD4) *via* interaction with p53 and induced the formation of NETs. Therefore, we put forward the hypothesis: Autophagy regulates PAD4-mediated NETs formation through the ATG7/p53 complex, which is an important mechanism for the self-remission of gouty arthritis. To verify this hypothesis, a series of studies, including clinicopathological investigation, *in vitro* and *in vivo* experiments were arranged to probe the molecular mechanism of autophagy regulating NETs formation. This study will help to further elucidate the correlation between ATG7 and PAD4, to provide a novel strategy to improve the therapeutic efficacy of gouty arthritis.

## Materials and methods

Multiple materials and methods are available in the **Supplementary Materials and Methods**.

### Reagents

The antibodies used were as follows: for western blotting and immunohistochemistry, rabbit monoclonal anti-myeloperoxidase (MPO) antibody (Abcam, Cambridge, UK), rabbit monoclonal anti-citrullinated histone H3 (H3cit) antibody (Abcam, Cambridge, UK), rabbit monoclonal anti-p53 antibody (Abcam, Cambridge, UK), rabbit monoclonal anti-ATG7 antibody (Abcam, Cambridge, UK), mouse monoclonal anti-PAD4 antibody (Abcam, Cambridge, UK) and rabbit monoclonal anti-GAPDH antibody (Cell Signaling Technology, MA, USA) were used. For immunofluorescence, mouse monoclonal anti-MPO antibody (Abcam, Cambridge, UK), rabbit monoclonal anti-H3cit antibody (Abcam, Cambridge, UK), Rhodamine Red-X (RRX) goat anti-mouse IgG (H+L) and FITC-AffiniPure goat anti-rabbit IgG (H+L) (Jackson, PA, USA) were used. The following chemicals were also utilized: MSU crystals (*In vivo*Gen, CA, USA); SYTOX Green (Thermo Fisher Scientific, MA, USA); Rapamycin (RAPA) and 3-Methyladenine (3MA) (Sigma-Aldrich, MO, USA); Pifithrin-α (Sigma-Aldrich, MO, USA). The recombinant human proteins used included recombinant p53 and recombinant ATG7 (R& D system, MN, USA).

### Human serum specimens

This study enrolled 30 healthy donors and 30 patients with gouty arthritis from January 2019 to December 2019 at the Department of Endocrinology, Jinling Hospital, Nanjing University Medical School. The diagnosis of acute gouty arthritis conformed to the standard issued by American College of Rheumatology/European Alliance of Associations for Rheumatology (ACR/EULAR) in 2015, and the exclusion criteria were as follows: 1) Patients with diabetes and serious heart, lung, liver and kidney diseases; 2) Joint infection; 3) Other diseases involving joints, such as rheumatoid arthritis, traumatic arthritis, pigmented villonodular synovitis, etc; 4) Those who have received intra-articular drug injection treatment within the past 6 months; 5) Those who have received glucocorticoid treatment in the past 4 weeks; 6) Those who have used non-steroidal anti-inflammatory drugs within 2 weeks. The patient cohort comprised acute stage and remission stage. All patients provided written informed consent. Serum sample and synovial fluid of joint were separated by sterile operation and stored at – 80°C for a long time. The study protocol was approved by the Institutional Review Board of Nanjing University and complied with the Helsinki Declaration.

### Animal experiments

WT C57BL/6 mice were purchased from the Shanghai Experimental Animal Center. PAD4^−/−^ mice were purchased from GemPharmatech Co., Ltd (Nanjing, China). All mice housed in a controlled environment (12h daylight cycle, lights off at 18:00) with food and water ad libitum. Air pouches were created on the backs of 8-week-old male mice. 4ml of filtered air was injected, followed by an additional 4ml of filtered air after 3d. Three days after the second injection, 1ml MSU crystals (3mg/ml) or PBS alone was injected into the air pouches. RAPA and 3MA were injected into the air pouch 1h before MSU crystals injection. After 4h, 8h, 16h, 24h, and 48h, the pouch fluid was harvested by injecting 1ml PBS for subsequent research. Another well-established gout mouse model is the paw edema model. 20μl MSU crystals (20mg/ml) or PBS were injected subcutaneously into the paws of mice. The right paw was stimulated with PBS and the left with MSU crystals. RAPA and 3MA were injected 30min before MSU crystals injection. Edema was evaluated at intervals of 12h and calculated as the thickness difference between the right and left paws. All animals were anesthetized in chambers saturated with isoflurane and killed.

### Neutrophil isolation and *in vitro* NETs induction

Neutrophils were isolated from peripheral blood of patients with gouty arthritis and healthy donors using Polymorphprep™ (Axis-Shield PoC AS, OSLO, Norway). The lysis of red blood cells was performed using a hypotonic solution (Solarbio, Beijing, China) according to the manuscript. The neutrophils (5x10^6^ cells) seeded in 10cm culture plates were cultured in RPMI 1640 with 10% fetal bovine serum in a humidified 5% CO_2_ incubator at 37°C. As determined by Wright staining and FACS analysis, the final neutrophil suspensions contained fewer than 0.2% monocytes or lymphocytes. Neutrophil viability exceeded 92% after up to 6h in culture, as determined by trypan blue exclusion and by Annexin V/propidium iodide FACS analysis (**Supplementary Figure Sl**). Isolated neutrophils were incubated with MSU crystals (200μg/ml) for 6h. After incubation for 30min, 1h, 2h, 3h, 4h, and 6h, media containing MSU crystals were removed. Each well was carefully washed twice with 1mL PBS and resuspended in DMEM. The supernatant of each well was collected and centrifuged for 5min at 200 g at 4°C to remove whole cells and debris.

### Detection of supernatant and serum NETs

NETs in supernatant and serum were assayed by SYTOX Green fluorescence and PicoGreen staining. SYTOX Green is a membrane-impermeable DNA-binding dye that can be used to quantify NETs-DNA. At the end of the incubation with MSU crystals, neutrophils were incubated with SYTOX Green (5μM) for 30min at 37°C. Then, after washing with PBS, NETs were observed by fluorescence microscopy. The levels of NETs in serum and synovial fluid were measured with Quant-iT PicoGreen dsDNA assay kit (Thermo Fisher Scientific, MA, USA) and MPO-DNA complexes detection ELISA kit (Roche, Indianapolis, IN) according to the manufacturer’s instructions.

### Kinetic binding analysis by biolayer interferometry

Kinetic binding analysis by Biolayer Interferometry (BLI) was performed according to our previous report (17). According to the manufacturer’s protocol, recombinant ATG7 (R& D system, MN, USA) was immobilized on the Amine Reactive Second-generation (AR2G) biosensor. Various concentrations of p53 (R& D system, MN, USA) were applied in the mobile phase and an association between the immobilized and flowing proteins was detected. The binding rate constant (Kon), dissociation rate constant (Kdis), and dissociation equilibrium constant (KD) were obtained by curve fitting of the association and dissociation phases of sensorgrams using a heterogeneous ligand model.

### Statistical analysis

All statistical analyses were performed using SPSS version 22.0 (SPSS Inc., IL, USA). The data were presented as mean ± SD or n (%). Differences in continuous variables in two groups were determined by the Student’s t test, and differences in categorical variables were determined by the χ2 analysis or Fisher exact test. Group differences were compared with ANOVA tests for normally distributed variables, whereas nonparametric Mann–Whitney U test was performed for skewed parameters. Dunn’s *post hoc* test was used for pairwise multiple comparisons, and the Bonferroni correction for α error was applied. P-values <0.05 were considered statistically significant.

## Results

### Neutrophil infiltration and the formation of NETs are found in gouty arthritis

**Figure 1:**
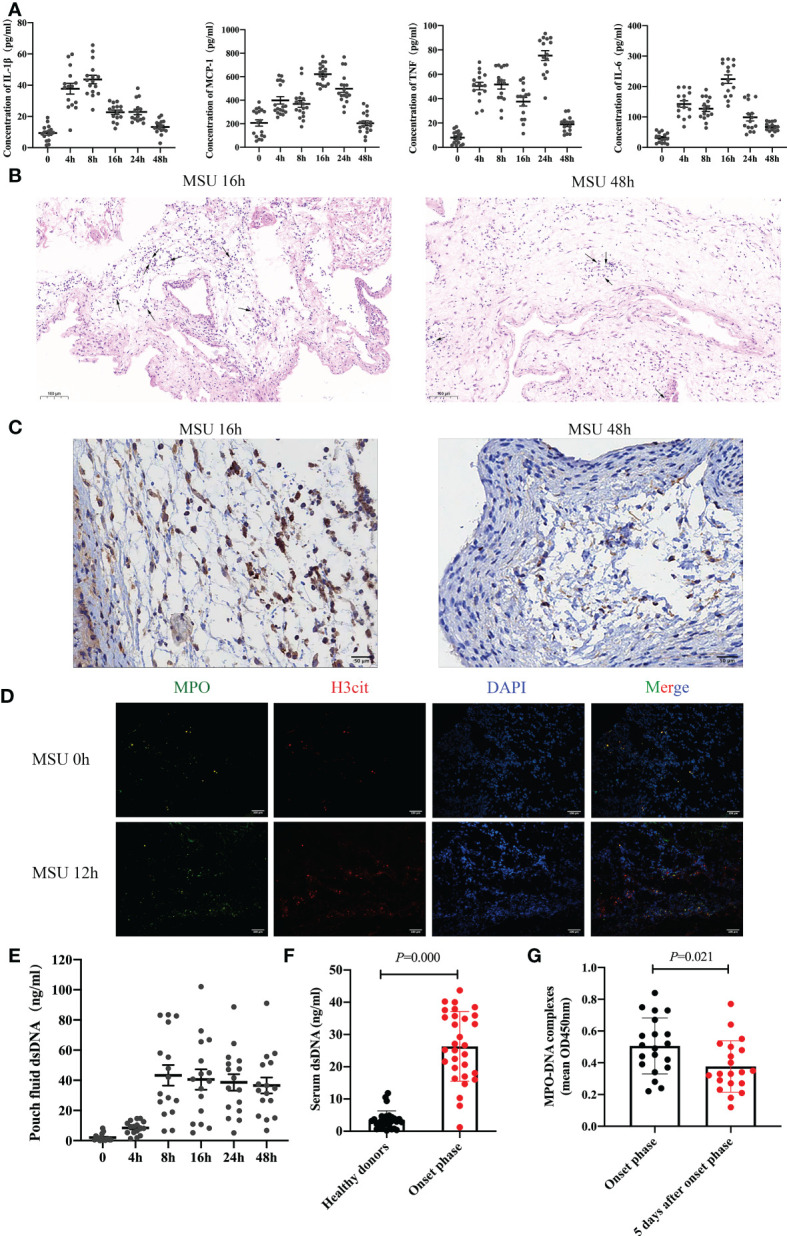
Neutrophil infiltration and the formation of NETs are found in gouty arthritis **(A)** The level of inflammatory cytokines (IL-1β, MCP-1, TNF, IL-6) in air pouch fluid from mice were detected at 0h, 4h, 8h, 16h, 24h, 48h by ELISA (n = 16 mice). **(B, C)** The result of HE staining **(B)** and Ly6G immunostaining **(C)** indicated that neutrophils infiltrated in the air pouch from mice. **(D)** The expression of NETs (MPO and H3cit positive) increased significantly at 12h with immunofluorescence assay on air pouch treated with MSU crystals. **(E)** After MSU crystals treatment, the dsDNA in pouch fluid from mice tested by ELISA increased significantly after 8h. **(F)** The serum dsDNA level was detected in 30 healthy donors and 30 patients with gouty arthritis at onset phase. The level of dsDNA at onset phase increased remarkably (*P* =0.000 vs healthy donors). **(G)** The level of MPO-DNA complexes in synovial fluid from patients (n=20) with gout during the onset phase could be clearly detected and significantly increased than 5 days after onset phase (*P* =0.000).

To observe the inflammatory milieu of gouty arthritis, we constructed a subcutaneous air pouch mouse model. MSU crystals were injected into the air pouch to detect the levels of inflammatory factors IL-1β, MCP-1, TNF, and IL-6 in the air pouch fluid at different time points (**Figure 1A**). The expression of inflammatory factors changed significantly. Interestingly, it peaked at 8-24h and decreased significantly at 48h. Subsequently, we removed the air pouch at each time point to investigate the expression of neutrophils. The results revealed significant neutrophil infiltration at 16h, which decreased significantly after 48h (**Figures 1B, C**). Thus, the expression of inflammatory factors and neutrophil infiltration decreased following the onset of gouty arthritis, given its self-limiting nature.

NETosis is significantly different from apoptosis or necrosis as a death pathway for neutrophils. Interestingly, neutrophils can deactivate pathogens by releasing extracellular structures of NETs composed of depolymerized chromatin and intracellular granule protein (18, 19). We took the air pouch at each time point to detect the expression of NETs. The results indicated that the expression of NETs increased significantly after MSU crystal treatment for 12h (**Figure 1D**). The decrease in neutrophil infiltration in gouty arthritis may be related to the formation of NETs. Further analysis confirmed that double-stranded DNA (dsDNA) in the air pouch fluid increased significantly after 8h (**Figure 1E**). Subsequently, we detected the serum dsDNA levels in 30 patients with gouty arthritis and 30 healthy donors. The level of dsDNA during the onset phase significantly increased (**Figure 1F**). To further investigate the connection between NETs and gout arthritis, synovial fluid was extracted from the swelling joint of 20 patients with gout. We analyzed the level of MPO-DNA complexes, which was also a common marker of NETs. The level of MPO-DNA complexes during the onset phase could be clearly detected and significantly increased than 5 days after onset phase (**Figure 1G**). These results collectively substantiate neutrophil infiltration and the formation of NETs in gouty arthritis.

### NETs induced by MSU crystals degrade inflammatory factors and alleviate the inflammatory response in gouty arthritis

**Figure 2:**
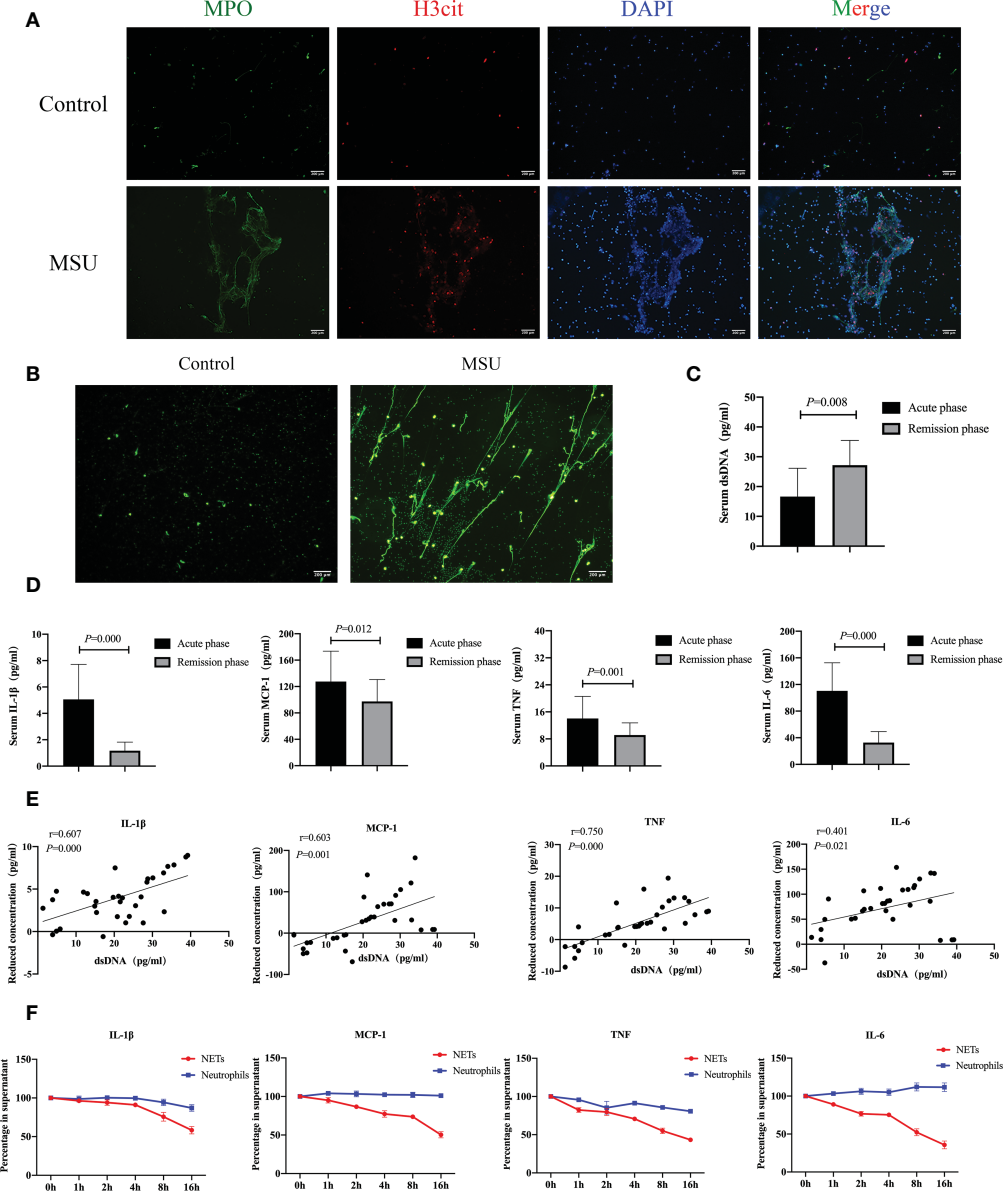
NETs induced by MSU crystals degrade inflammatory factors and alleviate the inflammatory response in gouty arthritis. **(A, B)** Neutrophils extracted from peripheral blood of patients with gouty arthritis could be induced to form NETs by MSU crystals *in vitro*. Immunofluorescence (MPO and H3cit positive) **(A)** and SYTOX Green fluorescence **(B)** indicated the formation of NETs. **C** The serum dsDNA detected by ELISA in acute stage and remission stage was tested respectively. The expression of dsDNA increased during emission stage (*P*=0.008). **(D)** The expression of serum inflammatory cytokines (IL-1β, MCP-1, TNF, IL-6) in acute stage and remission stage was detected respectively. IL-1β, MCP-1, TNF and IL-6 decreased significantly (*P*<0.05). **(E)** The dsDNA is proportional to the decrease of inflammatory cytokines (*P*<0.05). **(F)** Neutrophils extracted from patients with gouty arthritis were induced to form NETs by MSU crystals and added to the medium containing IL-1β, MCP-1, TNF and IL-6. The reduction of these inflammatory factors at different time points was analyzed by ELISA.

It is well-established that gouty arthritis is mainly caused by MSU crystals deposition. It has been considered that MSU crystals can be used as an inducer for the formation of NETs (20). We extracted neutrophils from the peripheral blood of patients with gouty arthritis. After neutrophils were treated with MSU crystals, significant NETs formation could be observed (**Figures 2A, B**). In addition, we divided the onset of gouty arthritis into acute and remission stages and then assessed the expression of serum dsDNA, IL-1β, MCP-1, TNF and IL-6. We found that dsDNA expression increased during gout remission, while IL-1β, MCP-1, TNF and IL-6 levels decreased significantly (**Figures 2C, D**). Interestingly, the expression of dsDNA is proportional to the decrease of inflammatory factors (**Figure 2E**). Subsequently, neutrophils extracted from patients with gouty arthritis were induced to form NETs by MSU crystals and added to the medium containing IL-1β, MCP-1, TNF and IL-6. The results showed that the reduction in these inflammatory factors was more pronounced than that of neutrophils alone (**Figure 2F**). Collectively, our studies suggest that MSU crystals can induce the formation of NETs, degrade inflammatory factors and alleviate the inflammatory response in gouty arthritis.

### MSU crystals result in profound molecular alterations and upregulate ATG7 expression in neutrophils

**Figure 3:**
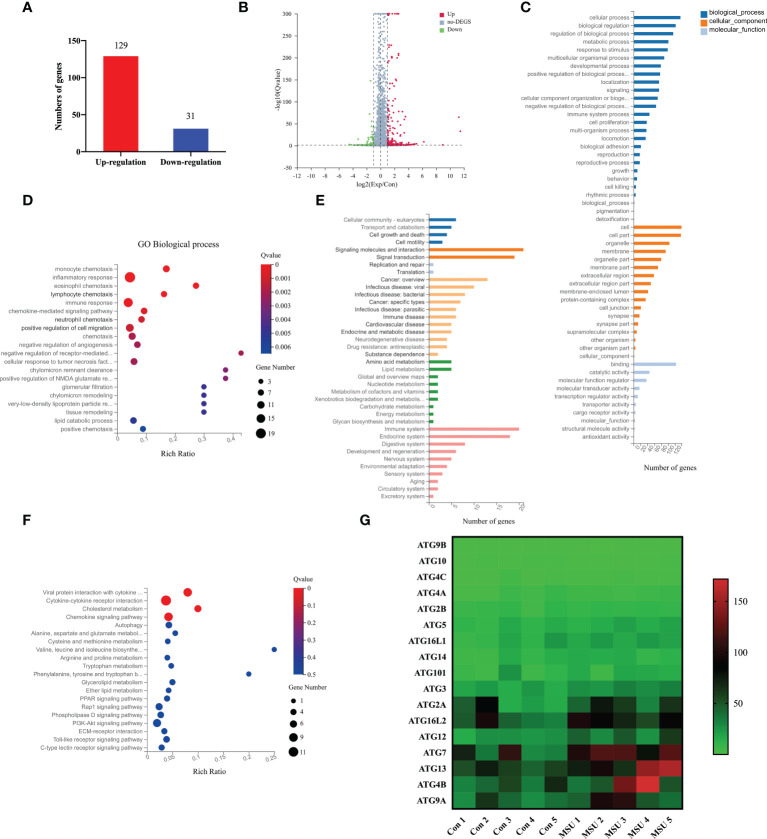
MSU crystals result in profound molecular alterations and upregulate ATG7 expression in neutrophils. **(A, B)** RNA sequencing indicated that 160 genes were differentially expressed in neutrophils under the stimulation of MSU crystals, including 129 upregulated genes and 31 downregulated genes. **(C)** Go analysis showed that DEGs were enriched in 53 terms, including 26 in the category of “biological processes”, 17 in “cell components”, and 10 in “molecular functions”. **(D)** DEGs were mainly enriched within the pathways of inflammatory response and immune response. **(E)** KEGG pathway analysis revealed that DGEs were abundant in the biological pathways including signal molecules, amino acid and lipid metabolism, immune system. **(F)** DEGs were mainly enriched in cytokine-cytokine receptor interaction, as well as chemokine signaling pathway and autophagy. **(G)** The heat map revealed that the expression of autophagy related genes was significantly changed after treatment with MSU crystals.

To identify the effects of MSU crystals on the function of neutrophils, we extracted neutrophils from five patients with gouty arthritis and treated them with MSU crystals. Then, RNA sequencing was performed to compare the genetic profiles of neutrophils cultured with or without MSU crystals for 6h. The results showed that 160 genes were differentially expressed in neutrophils after treatment with MSU crystals, including 129 up-regulated and 31 down-regulated genes (**Figures 3A, B**). Based on GO analysis, differentially expressed genes (DEGs) were mainly enriched in 53 terms, with 10 for “molecular functions”, 17 for “cell components”, and 26 terms for “biological processes” (**Figure 3C**). Furthermore, GO enrichment analysis of these DEGs showed significant enrichment in pathways of inflammatory response and immune response (**Figure 3D**). KEGG pathway analysis indicated that these DEGs were involved in various biological pathways, including amino acid and lipid metabolism, signal molecules, and the immune system (**Figure 3E**). These DEGs were remarkably enriched in the regulations of molecular functions, including cytokine-cytokine receptor interaction, as well as chemokine signaling pathway and autophagy (**Figure 3F**). Most notably, the expression of the autophagy related genes was significantly changed after treatment with MSU crystals (**Figure 3G**). These results indicated that MSU crystals result in profound molecular alterations in neutrophils that may be involved in NETs formation.

### MSU crystals stimulate neutrophil autophagy and induce the formation of NETs

**Figure 4:**
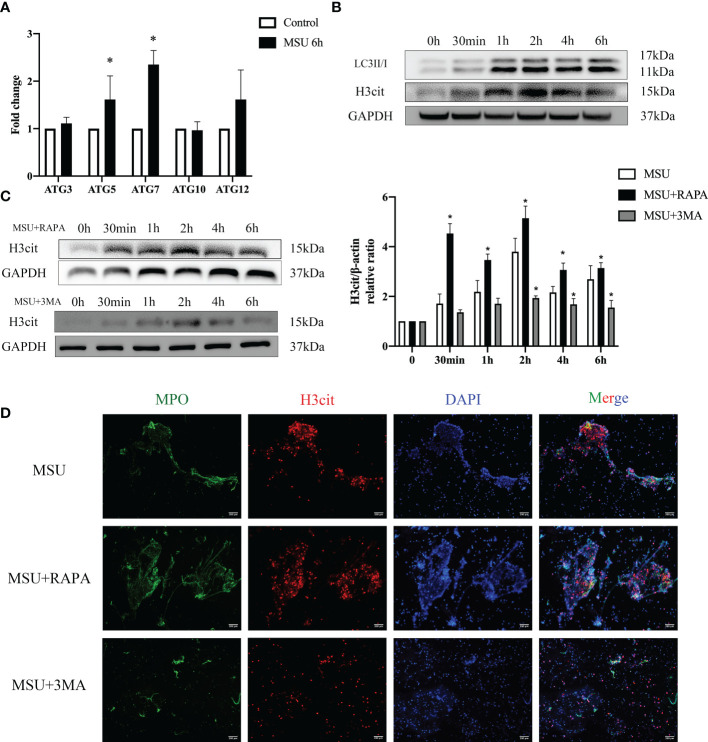
MSU crystals stimulate neutrophil autophagy and induce the formation of NETs. **(A, B)** MSU crystals enhanced the autophagy of neutrophils. The expression of LC3II/I, ATG5 and ATG7 increased significantly (*P < 0.05) valued by real-time PCR and western blotting assays. **(B)** The expression of H3cit increased significantly in neutrophils treated with MSU crystals for 6h in the western blotting assay. **(C)** The activators (RAPA) and inhibitors (3MA) of autophagy correspondingly changed the expression of H3cit (*P < 0.05). **(D)** The formation of NETs (MPO and H3cit positive) increased or decreased with the addition of RAPA and 3MA using immunofluorescence assay.

The results of RNA-seq showed that MSU crystals enhanced the autophagy of neutrophils. Western blot and qPCR analysis demonstrated that the expression of LC3II/I, autophagy related protein 5 (ATG5) and ATG7 increased significantly (**Figures 4A, B**). However, it remains unclear whether autophagy induced by MSU crystals is related to the formation of NETs. The formation of NETs usually depends on the capability of PAD4, which is a major regulator of NETs formation via citrullination of histone H3, causing chromatin depolymerization and release (21, 22). Therefore, H3cit is the most common maker of NETs. Accordingly, we first stimulated neutrophils with MSU crystals for 6h resulting in increased expression of H3cit (**Figure 4B**). At the same time, when RAPA, an activator of autophagy, was added to the culture supernatant, the expression of H3cit increased rapidly. In contrast, the addition of autophagy inhibitor 3MA slowed down the expression of H3cit (**Figure 4C**). The immunofluorescence assay showed that the formation of NETs increased or decreased with the addition of RAPA and 3MA (**Figure 4D**). Therefore, we hypothesized that MSU crystals could stimulate neutrophil autophagy and induce the formation of NETs.

### ATG7 upregulation in neutrophils promotes the expression of PAD4 via interactions with p53 and induces the formation of NETs

**Figure 5:**
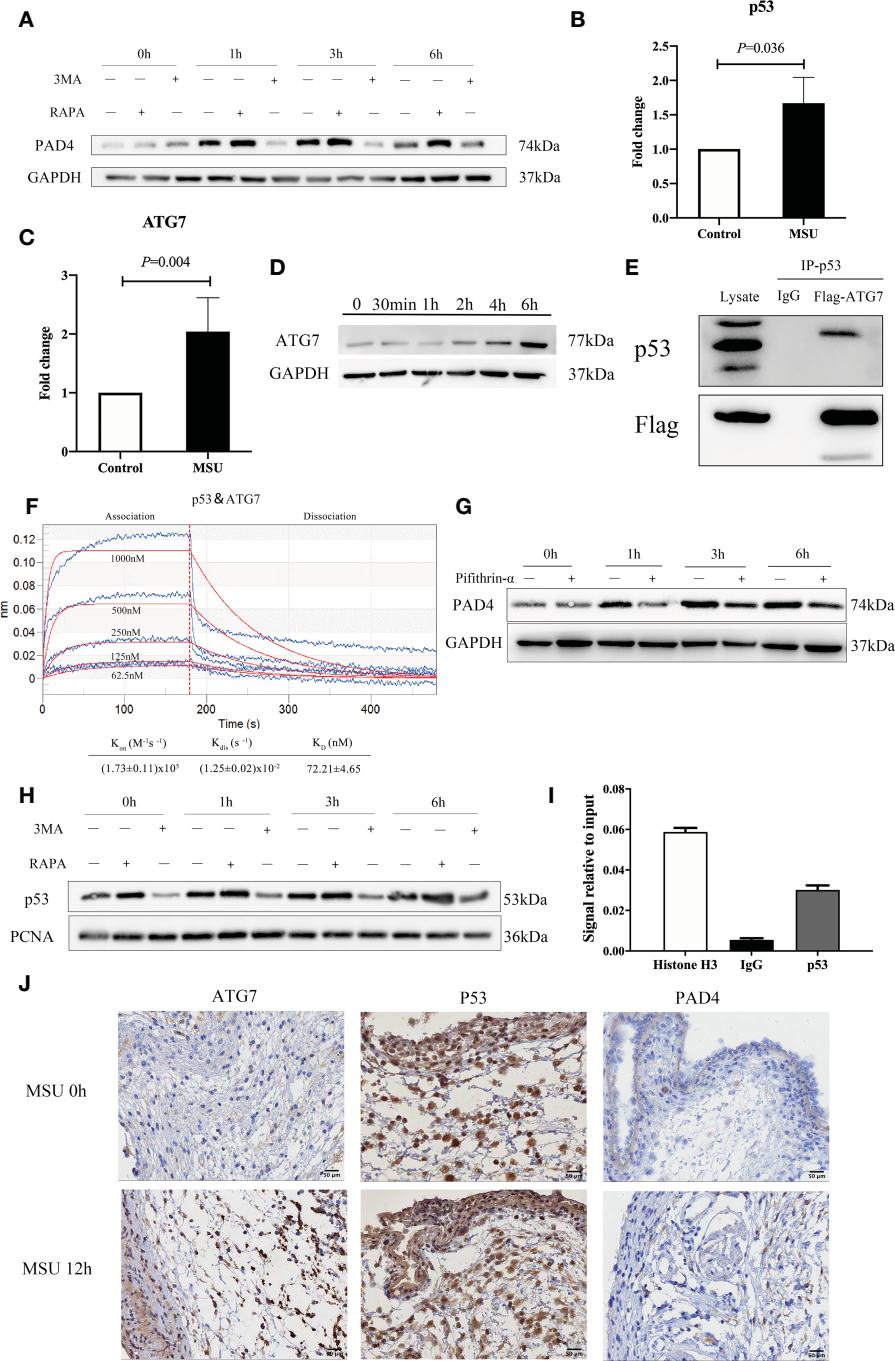
ATG7 upregulation in neutrophils promotes the expression of PAD4 via interactions with p53 and induces the formation of NETs. **(A)** The expression of PAD4 increased or decreased accordingly with the treatment of RAPA and 3MA in neutrophils. **(B)** p53 was increased in neutrophils with the treatment of MSU crystals in the RT−qPCR assay (*P* = 0.036). **(C)** The expression of ATG7 gene was increased more than two times induced by MSU crystals in RNA sequencing analysis (*P*=0.004). **(D)** Western blotting assays showed that the ATG7 levels in neutrophils increased remarkably in the treatment of MSU crystals. **(E)** ATG7 were identified to be associated with p53 by an immune coprecipitation assay. **(F)** There was a relative intense affinity between ATG7 and p53 in BLI assay. **(G)** Pifithrin-α (p53 inhibitor) could significantly reduce the expression of PAD4 in neutrophils. **(H)** RAPA and 3MA correspondingly regulated the expression of p53 in the nucleus of neutrophils. **(I)** The DNA binding of p53 was much higher than IgG in ChIP-PCR assay. **(J)** ATG7 and PAD4 increased significantly after 12 hours of MSU treatment in Immunohistochemistry. While the change of p53 was not obvious.

Little is currently known about how neutrophil autophagy induced by MSU crystals regulates the formation of NETs. After neutrophils were treated with MSU crystals, the expression of PAD4 increased. Subsequently, when RAPA and 3MA were added, the expression of PAD4 increased or decreased accordingly (**Figure 5A**). It has been established that p53 as a transcription factor exerts effects through interaction with autophagy-related proteins (23). Therefore, we speculated that autophagy-related proteins could bind to p53 and play a synergistic role. RNA-seq showed that p53 was also increased after MSU crystals stimulated neutrophils (**Figure 5B**). Most notably, the expression of ATG7 was enhanced more than two-fold higher after treatment with MSU crystals (**Figure 5C**). This finding was confirmed by subsequent western blotting (**Figure 5D**). As shown in **Figure 5E**, p53 was found to bind with ATG7 through the co-immunoprecipitation assay.

As a further measure of interactions between p53 and ATG7, we conducted a Biolayer Interferometry (BLI) experiment. Based on the results of **Figure 5F**, we evaluated the binding affinity of ATG7 peptides at different concentrations of p53 peptides (62.5, 125.0, 250.0, 500.0, and 1000 μM). For binding of p53 to ATG7, calculated values were as follows: Kon = (1.73 ± 0.11) x10^5^M^−1^S^−1^ for the association phase, Kdis = (1.25 ± 0.02) x10^-2^S^−1^ for the dissociation phase, an overall dissociation constant KD = 72.21 ± 4.65nM. These results indicated an intense affinity between p53 and ATG7.

As a transcription factor, p53 regulates the transcription of many proteins. We performed inhibition assay using Pifithrin-α, a p53 inhibitor, and the results indicated that treatment of Pifithrin-α decreased the expression of PAD4 (**Figure 5G**). Subsequently, we extracted the nuclear protein and detected the expression of p53 in the nucleus after treatment with MSU crystals. RAPA and 3MA regulated the expression of p53 in the nucleus (**Figure 5H**). In a subsequent experiment, we assessed the effect of p53 on DNA binding to the promoter of PAD4 using the ChIP-PCR assay. As shown in **Figure 5I**, the DNA binding of p53 was much higher, indicating that p53 was the transcription factor responsible for PAD4 expression. In addition, immunohistochemistry (IHC) staining was used to detect the expression of ATG7, p53 and PAD4 in the air pouch. The results revealed that ATG7 and PAD4 increased significantly at 12h, while the change in p53 was not obvious (**Figure 5J**). These results collectively suggest that ATG7 produced by neutrophils stimulated by MSU crystals work synergistically with p53 to enter the nucleus, which promotes the expression of PAD4 and then induces the formation of NETs.

### The autophagy of neutrophils regulates the severity of gouty arthritis via the formation of NETs

**Figure 6:**
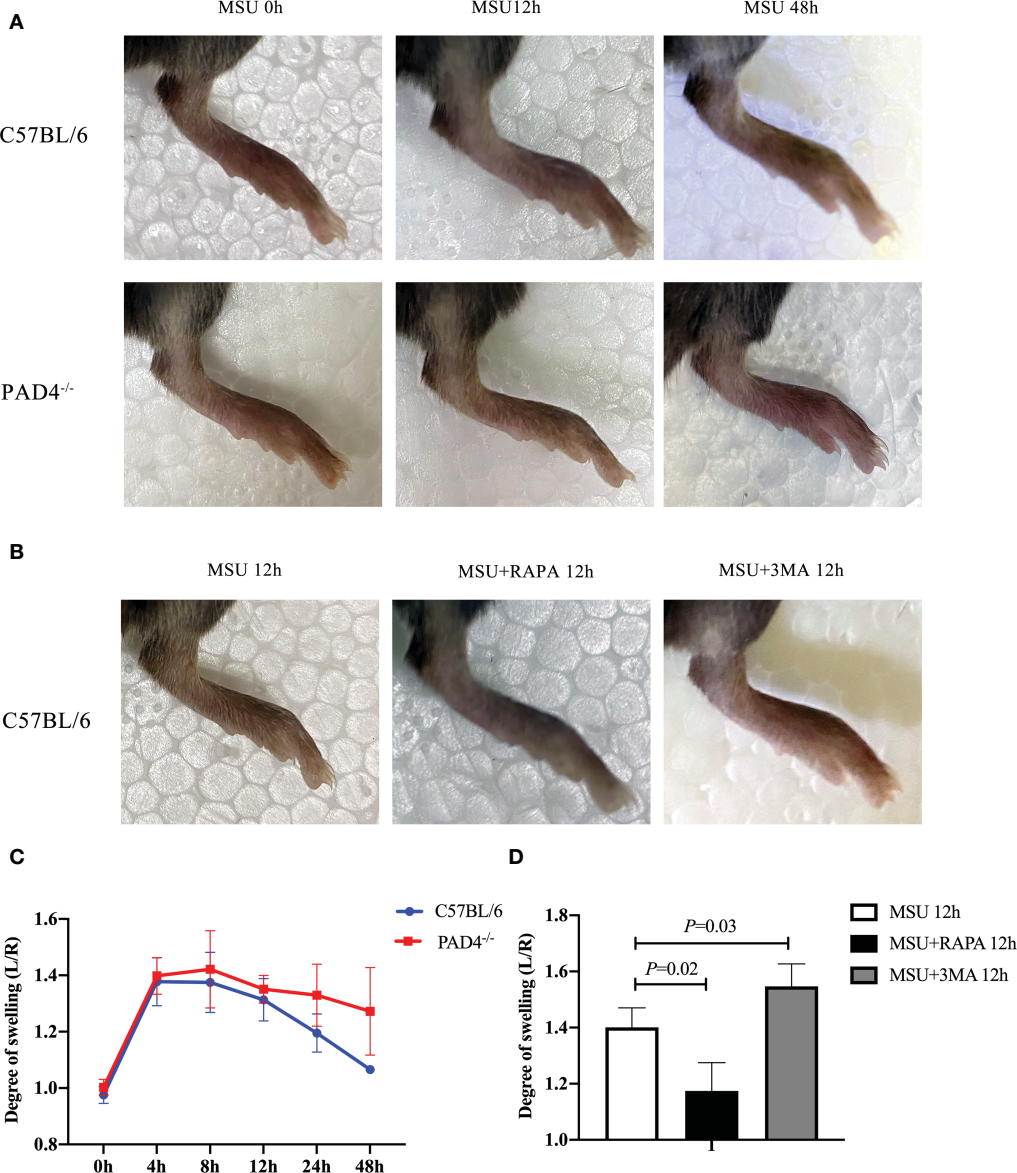
The autophagy of neutrophils regulates the severity of gouty arthritis via the formation of NETs. **(A, C)** Foot swelling was used to assess the severity of gout in wild-type and PAD4^-/-^ mice. Edema was evaluated at intervals of 12h and calculated as the thickness difference between the right and left paws. Paw edema caused by MSU crystals was self-limited in wild-type mice within 48h. While the degree of swelling increased significantly in PAD4^-/-^ mice. **(B, D)** RAPA and 3MA were injected with MSU crystals at the same time. The degree of foot swelling decreased or increased accordingly (*P* < 0.05).

To confirm the role of NETs in gouty arthritis, we constructed PAD4^-/-^ mice. Foot swelling was used to assess the severity of gout. MSU crystals injection resulted in paw edema, which exhibited a self-limiting course in C57BL/6 mice (**Figure 6A, C**). However, in PAD4^-/-^ mice, the degree of swelling did not decrease significantly within 48 hours. Subsequently, RAPA and 3MA were injected with MSU crystals, leading to a decrease and increase in foot swelling, respectively (**Figures 6B, D**). These observations indicate that the autophagy of neutrophils regulates the severity of gouty arthritis via the formation of NETs. Collectively, the autophagy of neutrophils regulates the formation of NETs, degrades inflammatory factors, and accounts for the self-limiting course during gouty arthritis.

## Discussion

**Figure 7:**
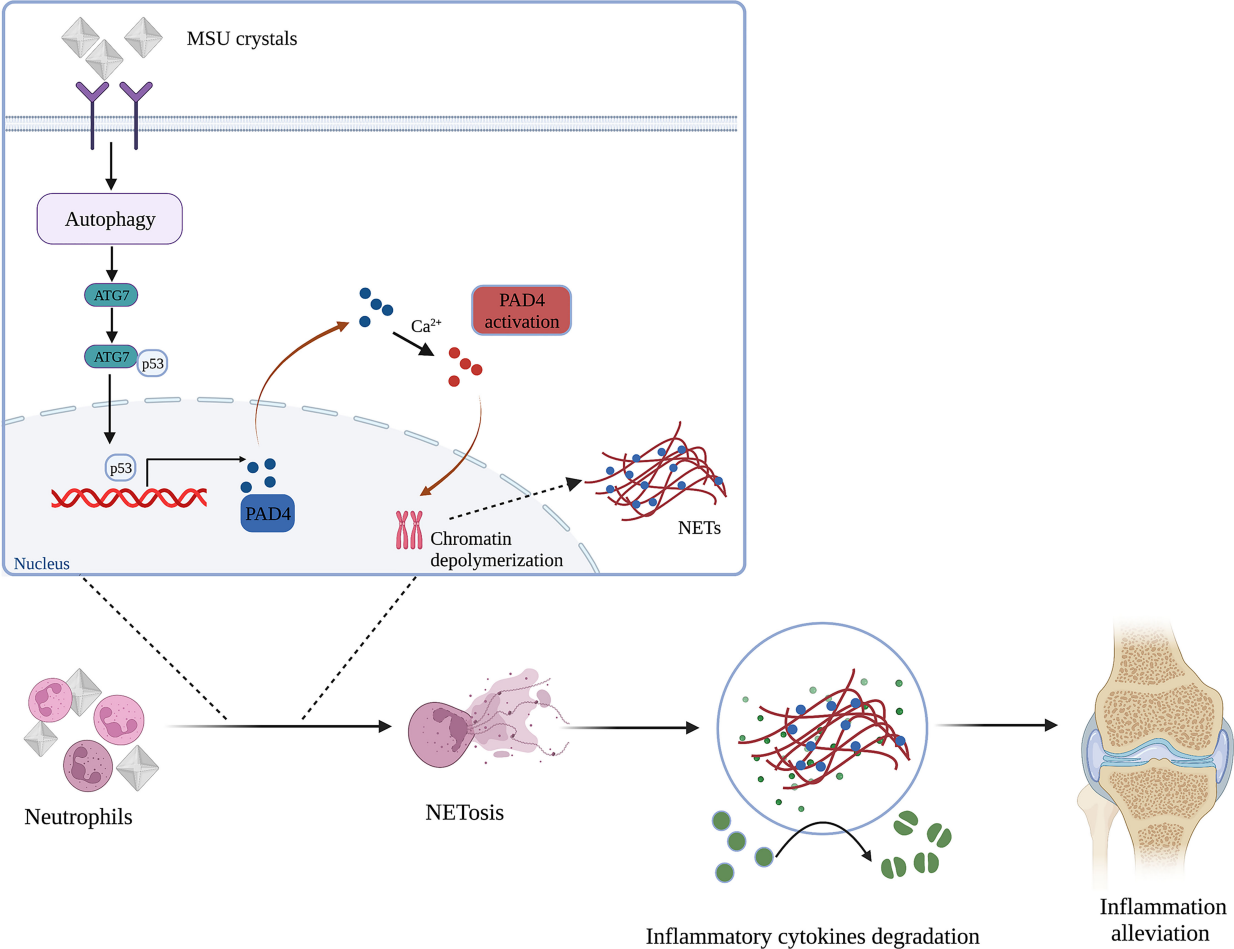
Schematic representation of the mechanism for NETs induced by neutrophil autophagy promoting inflammation remission in gouty arthritis (Created with BioRender.com).

It is well-established that gouty arthritis is an aseptic inflammatory reaction induced by MSU crystals. Furthermore, increasing evidence shows that neutrophil infiltration may play a vital role in the progression of gouty arthritis (24, 25). However, the causal relationships among MSU crystals, neutrophils and inflammatory response in gouty arthritis remain unclear. Our work provides compelling evidence that MSU crystals can induce the formation of NETs by infiltrating neutrophils to degrade inflammatory cytokines. Interestingly, MSU crystals can induce neutrophil autophagy and expression of ATG7, which can bind to p53 and promote PAD4 transcription, leading to the formation of NETs (**Figure 7**). Our studies identified a novel mechanism whereby MSU crystals promote neutrophil autophagy and induce the formation of NETs.

Recent evidence has emerged that NETs might play an important role in noninfectious diseases. The formation of NETs can be triggered by endogenous stimuli and microorganisms, such as crystals, bacteria, viruses, fungi and immune complexes. It has been established that MSU crystals can induce the formation of NETs (26, 27). IL-1β reportedly plays a key role in the recruitment of neutrophils and the process of NETs generation (28). In addition, MSU crystals-induced NETosis may be mediated by ROS via RIPK1-RIPK3-MLKL signaling (29). In the current study, we demonstrated that MSU crystals could recruit neutrophils leading to the aggregation and formation of NETs. We further revealed that the effect of MSU crystals on neutrophils could change their cell phenotype and biological function. Among these DEGs, a number of DEGs were screened as candidate genes for further investigation, including CCL7, CD276, and PRLR. However, these DEGs were unrelated to the regulatory effect of MSU crystals on neutrophils (data not shown). Based on literature review and bioinformatics analysis, ATG7 was selected as the target gene in our study. It is well-established that ATG7 is a key protein in the autophagy pathway. MSU crystals may promote autophagy in neutrophils related to the formation of NETs.

Autophagy is a critical cellular process through which cell organelles are degraded in the lysosome to protect cells and organisms from stressors (30). It is worth noting that autophagy also plays an important role in alleviating the inflammatory response to gouty arthritis. MSU crystals stimulation of NLRP3 inflammasome activation is accompanied by increased autophagy activity, which can negatively regulate NLRP3 and limit the generation of IL-1β (31). In contrast, adding autophagy agonists to rats with gouty arthritis induced by MSU crystals can reduce joint swelling and the expression of cytokines (15). Limited information, however, is available about the mechanism of autophagy in gouty arthritis. Herein, we revealed that MSU crystals could induce neutrophil autophagy *in vitro* and *in vivo*. In addition, RNA-seq suggested that autophagy occurred in neutrophils after MSU crystals stimulation. Our findings suggest that once neutrophils are activated, degranulation occurs, and autophagy regulates this process, which is related to the formation of NETs.

In neutrophils, the activation of autophagy accelerates the formation of NETs, while autophagy inhibitors can inhibit the release of NETs, which fully proves that there is a definite relationship between autophagy and the formation of NETs (32). It is well-established that mTOR is an important node in autophagy regulation. Inhibiting the mTOR signaling axis can promote neutrophil autophagy and accelerate NETs generation. In this study, we demonstrated that ATG7 produced by neutrophils stimulated with MSU crystals work synergistically with p53 to enter the nucleus and induce the formation of NETs. Although the activation of autophagy can produce many autophagy-related proteins, we reason that only ATG7 attributes to the generation of NETs. Indeed, further investigation is warranted to increase the robustness of our findings. Nevertheless, we established that ATG7 expression induced by MSU crystals is related to NETs generation.

Our study identified the molecular mechanisms underlying the upregulated expression of ATG7 and its role in inducing PAD4. It is well-established that the formation of NETs relies on the role of PAD4. Structurally, PAD4 is a calcium-dependent enzyme with five calcium-binding sites and two folded domains (33). During the formation of NETs, histones undergo citrullination and promote chromosome depolymerization. PAD4 is the main enzyme involved in histone citrullination (34). However, the mechanism regulating PAD4 production remains uncertain. As a transcription factor, p53 regulates DNA damage repair and apoptosis-related gene transcription (35). We hypothesized that p53 might regulate the transcription of PAD4. The impact of p53 on DNA binding of PAD4 promoter was much higher than observed during ChIP-PCR. Interestingly, autophagy regulates the expression of p53 in the nucleus of neutrophils. BLI assays indicated a strong affinity between ATG7 and p53. Collectively, our studies suggest that ATG7 produced by neutrophils stimulated by MSU crystals can work synergistically with p53 to enter the nucleus, which promotes the expression of PAD4 and induces the formation of NETs.

This study has several limitations. We used a global knock-out mouse model and focused on the local reactions with paw edema model. Besides, the results were not directly verified in the joint synovium of gout patients. In addition, we mainly explored the role of ATG7, but this does not exclude other autophagy-related proteins can have an additive effect on the formation of NETs, such as ATG5 or autophagy related protein 12 (ATG12).

Our study provided a novel strategy for the therapy of gouty arthritis. We substantiated the effects of NETs induced by MSU crystals on the degradation of inflammatory factors. Promoting the formation of NETs or reducing the degradation of NETs in the affected joint may be beneficial in alleviating inflammation in the acute stage of gout. Consistent with the literature, our study showed that autophagy is critically involved in the process of gout. However, merely activating autophagy may not be effective and even lead to the progression of gout. Thus, future studies should focus on specific subsets of neutrophils with unique roles in affected joints, providing critical information for gouty arthritis.

## Data availability statement

The data presented in the study are deposited in the NCBI-bioproject repository, accession number PRJNA893078.

## Ethics statement

The studies involving human participants were reviewed and approved by The Ethics Committee of Nanjing University. The patients/participants provided their written informed consent to participate in this study. The animal study was reviewed and approved by The Ethics Committee of Nanjing University. Written informed consent was obtained from the individual(s) for the publication of any potentially identifiable images or data included in this article.

## Author contributions

HL and JS conceptualized the study. SH, YW and JS performed the experiments. SL and WG analyzed data. SH and JS wrote the manuscript. All authors contributed to the article and approved the submitted version.

## Supplementary material

Supplementary Figure 1 Measurement of the purity and viability of isolated neutrophil. **(A)** Wright staining and **(B)** FACS analysis showed that the final neutrophil suspensions contained fewer than 0.2% monocytes or lymphocytes. **(C)** Neutrophil viability exceeded 92% after up to 6h in culture, as determined by Annexin V/propidium iodide FACS analysis.

## References

[1] Dalbeth N Gosling AL Gaffo A Abhishek A. Gout. Lancet (2021) 397(10287):1843–55. doi: 10.1016/S0140-6736(21)00569-9 33798500

[2] Cabau G Crisan TO Kluck V Popp RA Joosten LAB. Urate-induced immune programming: consequences for gouty arthritis and hyperuricemia. Immunol Rev (2020) 294(1):92–105. doi: 10.1111/imr.12833 31853991 PMC7065123

[3] Dehlin M Jacobsson L Roddy E. Global epidemiology of gout: prevalence, incidence, treatment patterns and risk factors. Nat Rev Rheumatol (2020) 16(7):380–90. doi: 10.1038/s41584-020-0441-1 32541923

[4] Liu R Han C Wu D Xia X Gu J Guan H. Prevalence of hyperuricemia and gout in mainland China from 2000 to 2014: a systematic review and meta-analysis. BioMed Res Int (2015) 2015:762820. doi: 10.1155/2015/762820 26640795 PMC4657091

[5] Anderson IJ Davis AM Jan RH. Management of gout. JAMA (2021) 326(24):2519–20. doi: 10.1001/jama.2021.19763 34962547

[6] Richette P Bardin T. Gout. Lancet (2010) 375(9711):318–28. doi: 10.1016/S0140-6736(09)60883-7 19692116

[7] Stamp LK Dalbeth N. Prevention and treatment of gout. Nat Rev Rheumatol (2019) 15(2):68–70. doi: 10.1038/s41584-018-0149-7 30546062

[8] Busso N So A. Mechanisms of inflammation in gout. Arthritis Res Ther (2010) 12(2):206. doi: 10.1186/ar2952 20441605 PMC2888190

[9] Szekanecz Z Szamosi S Kovacs GE Kocsis E Benko S. The Nlrp3 inflammasome - interleukin 1 pathway as a therapeutic target in gout. Arch Biochem Biophys (2019) 670:82–93. doi: 10.1016/j.abb.2019.01.031 30710503

[10] So AK Martinon F. Inflammation in gout: mechanisms and therapeutic targets. Nat Rev Rheumatol (2017) 13(11):639–47. doi: 10.1038/nrrheum.2017.155 28959043

[11] Galozzi P Bindoli S Doria A Oliviero F Sfriso P. Autoinflammatory features in gouty arthritis. J Clin Med (2021) 10(9). doi: 10.3390/jcm10091880 PMC8123608 33926105

[12] Steiger S Harper JL. Mechanisms of spontaneous resolution of acute gouty inflammation. Curr Rheumatol Rep (2014) 16(1):392. doi: 10.1007/s11926-013-0392-5 24343224

[13] Cumpelik A Ankli B Zecher D Schifferli JA. Neutrophil microvesicles resolve gout by inhibiting C5a-mediated priming of the inflammasome. Ann Rheum Dis (2016) 75(6):1236–45. doi: 10.1136/annrheumdis-2015-207338 PMC4893114 26245757

[14] Lou D Zhang X Jiang C Zhang F Xu C Fang S. 3beta,23-Dihydroxy-12-Ene-28-Ursolic acid isolated from cyclocarya paliurus alleviates Nlrp3 inflammasome-mediated gout *Via* Pi3k-Akt-Mtor-Dependent autophagy. Evid Based Complement Alternat Med (2022) 2022:5541232. doi: 10.1155/2022/5541232 35047046 PMC8763513

[15] Chen H Zheng S Wang Y Zhu H Liu Q Xue Y. The effect of resveratrol on the recurrent attacks of gouty arthritis. Clin Rheumatol (2016) 35(5):1189–95. doi: 10.1007/s10067-014-2836-3 25451618

[16] Mitroulis I Kambas K Chrysanthopoulou A Skendros P Apostolidou E Kourtzelis I. Neutrophil extracellular trap formation is associated with il-1beta and autophagy-related signaling in gout. PloS One (2011) 6(12):e29318. doi: 10.1371/journal.pone.0029318 22195044 PMC3241704

[17] Shen J Zhai J You Q Zhang G He M Yao X. Cancer-associated fibroblasts-derived Vcam1 induced by h. pylori infection facilitates tumor invasion in gastric cancer. Oncogene (2020) 39(14):2961–74. doi: 10.1038/s41388-020-1197-4 32034307

[18] Papayannopoulos V. Neutrophil extracellular traps in immunity and disease. Nat Rev Immunol (2018) 18(2):134–47. doi: 10.1038/nri.2017.105 28990587

[19] Sollberger G Tilley DO Zychlinsky A. Neutrophil extracellular traps: the biology of chromatin externalization. Dev Cell (2018) 44(5):542–53. doi: 10.1016/j.devcel.2018.01.019 29533770

[20] Holmes CL Shim D Kernien J Johnson CJ Nett JE Shelef MA. Insight into neutrophil extracellular traps through systematic evaluation of citrullination and peptidylarginine deiminases. J Immunol Res (2019) 2019:2160192. doi: 10.1155/2019/2160192 30993117 PMC6434303

[21] Lewis HD Liddle J Coote JE Atkinson SJ Barker MD Bax BD. Inhibition of Pad4 activity is sufficient to disrupt mouse and human net formation. Nat Chem Biol (2015) 11(3):189–91. doi: 10.1038/nchembio.1735 PMC4397581 25622091

[22] Wang Y Wysocka J Sayegh J Lee YH Perlin JR Leonelli L. Human Pad4 regulates histone arginine methylation levels *Via* demethylimination. Science (2004) 306(5694):279–83. doi: 10.1126/science.1101400 15345777

[23] White E. Autophagy and P53. Cold Spring Harb Perspect Med (2016) 6(4):a026120. doi: 10.1101/cshperspect.a026120 27037419 PMC4817743

[24] Yin C Liu B Li Y Li X Wang J Chen R. Il-33/St2 induces neutrophil-dependent reactive oxygen species production and mediates gout pain. Theranostics (2020) 10(26):12189–203. doi: 10.7150/thno.48028 PMC7667675 33204337

[25] Goldberg EL Asher JL Molony RD Shaw AC Zeiss CJ Wang C. Beta-hydroxybutyrate deactivates neutrophil Nlrp3 inflammasome to relieve gout flares. Cell Rep (2017) 18(9):2077–87. doi: 10.1016/j.celrep.2017.02.004 PMC5527297 28249154

[26] Sil P Hayes CP Reaves BJ Breen P Quinn S Sokolove J. P2y6 receptor antagonist Mrs2578 inhibits neutrophil activation and aggregated neutrophil extracellular trap formation induced by gout-associated monosodium urate crystals. J Immunol (2017) 198(1):428–42. doi: 10.4049/jimmunol.1600766 27903742

[27] Schauer C Janko C Munoz LE Zhao Y Kienhofer D Frey B. Aggregated neutrophil extracellular traps limit inflammation by degrading cytokines and chemokines. Nat Med (2014) 20(5):511–7. doi: 10.1038/nm.3547 24784231

[28] Hugle T Krenn V. [Histopathophysiology of gout]. Ther Umsch (2016) 73(3):137–40. doi: 10.1024/0040-5930/a000769 27008445

[29] Desai J Kumar SV Mulay SR Konrad L Romoli S Schauer C. Pma and crystal-induced neutrophil extracellular trap formation involves Ripk1-Ripk3-Mlkl signaling. Eur J Immunol (2016) 46(1):223–9. doi: 10.1002/eji.201545605 26531064

[30] Mizushima N Levine B. Autophagy in human diseases. N Engl J Med (2020) 383(16):1564–76. doi: 10.1056/NEJMra2022774 33053285

[31] Shi CS Shenderov K Huang NN Kabat J Abu-Asab M Fitzgerald KA. Activation of autophagy by inflammatory signals limits il-1beta production by targeting ubiquitinated inflammasomes for destruction. Nat Immunol (2012) 13(3):255–63. doi: 10.1038/ni.2215 PMC4116819 22286270

[32] Itakura A McCarty OJ. Pivotal role for the mtor pathway in the formation of neutrophil extracellular traps *Via* regulation of autophagy. Am J Physiol Cell Physiol (2013) 305(3):C348–54. doi: 10.1152/ajpcell.00108.2013 PMC3742850 23720022

[33] Slade DJ Subramanian V Thompson PR. Pluripotency: citrullination unravels stem cells. Nat Chem Biol (2014) 10(5):327–8. doi: 10.1038/nchembio.1504 PMC4632640 24743255

[34] Li P Li M Lindberg MR Kennett MJ Xiong N Wang Y. Pad4 is essential for antibacterial innate immunity mediated by neutrophil extracellular traps. J Exp Med (2010) 207(9):1853–62. doi: 10.1084/jem.20100239 PMC2931169 20733033

[35] Sullivan KD Galbraith MD Andrysik Z Espinosa JM. Mechanisms of transcriptional regulation by P53. Cell Death Differ (2018) 25(1):133–43. doi: 10.1038/cdd.2017.174 PMC5729533 29125602

