# Supplementary figures and images for: Neutrophil autophagy induced by monosodium urate crystals facilitates neutrophil extracellular traps formation and inflammation remission in gouty arthritis
Source: Front Endocrinol (Lausanne). 2023 Sep 22;14:1071630. doi: 10.3389/fendo.2023.1071630 (PMC10557066; doi:10.3389/fendo.2023.1071630)

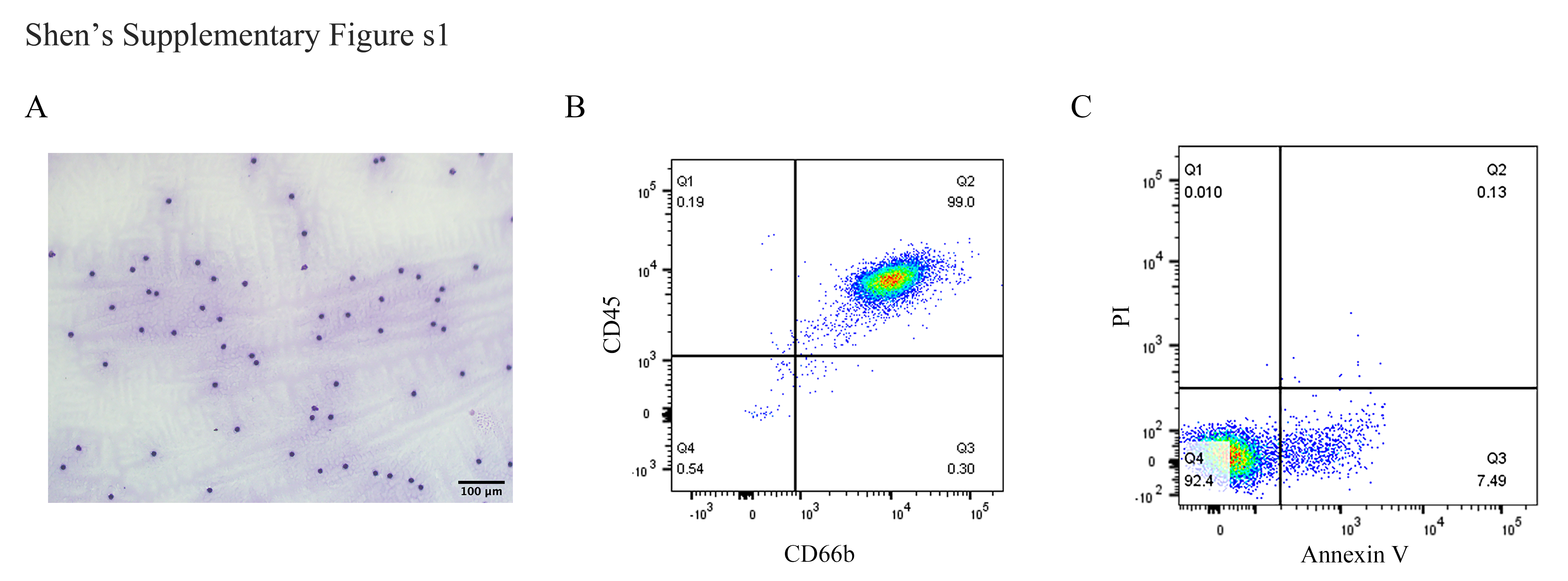

Supplement: Supplementary Figure 1 — Measurement of the purity and viability of isolated neutrophil. (A) Wright staining and (B) FACS analysis showed that the final neutrophil suspensions contained fewer than 0.2% monocytes or lymphocytes. (C) Neutrophil viability exceeded 92% after up to 6h in culture, as determined by Annexin V/propidium iodide FACS analysis. [file Image_1.tif]

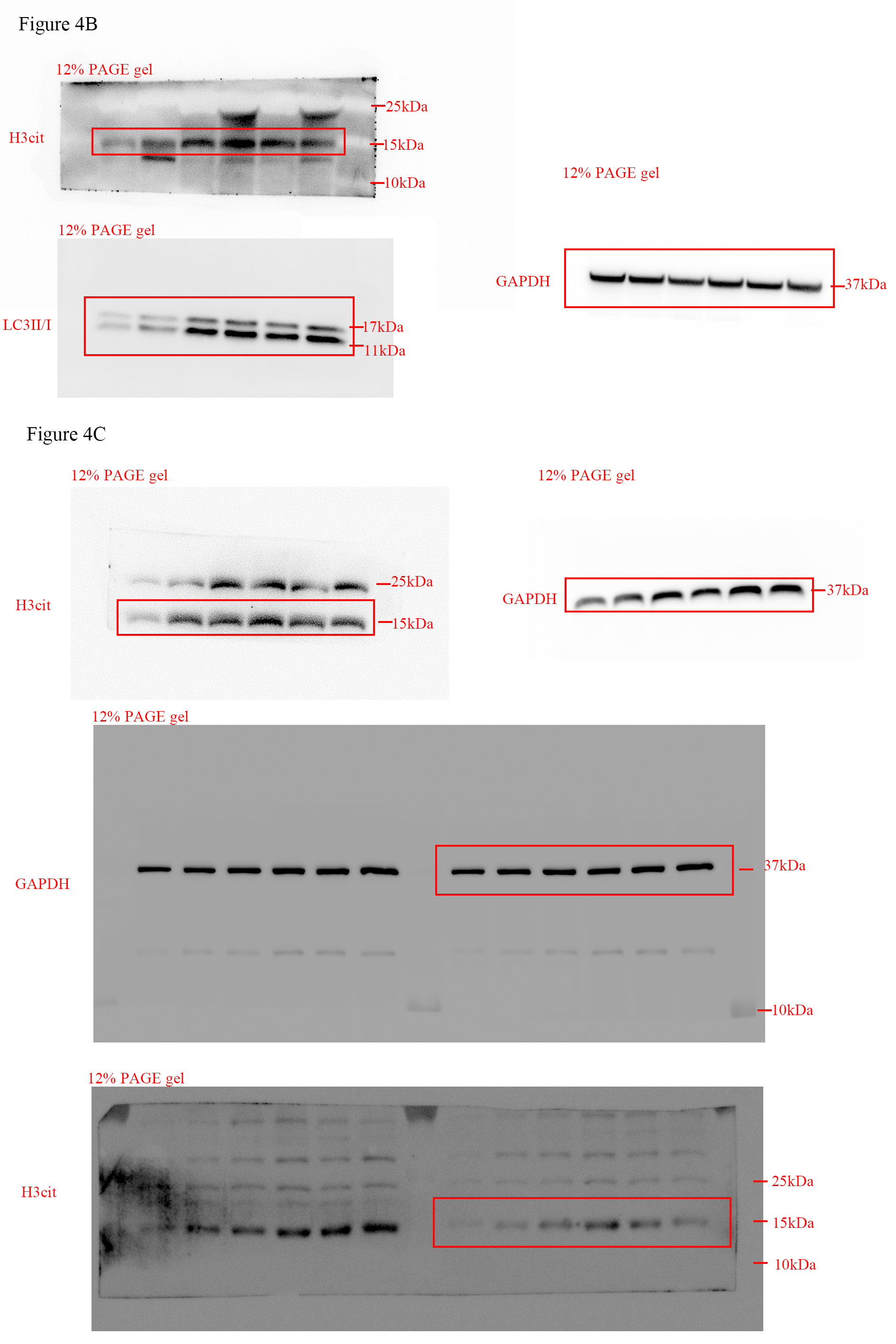

Supplement: Supplementary file 2 [file Image_2.tif]

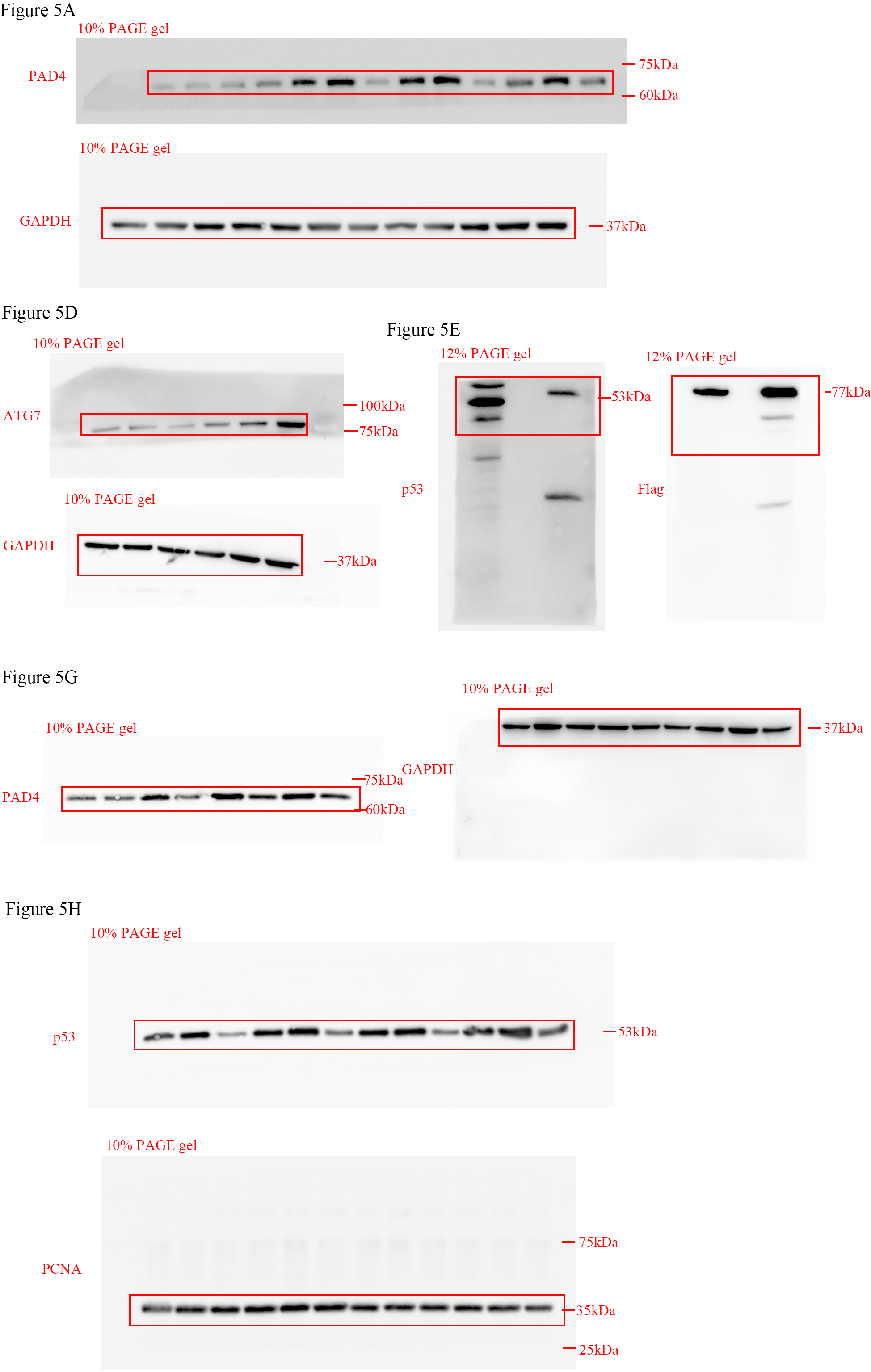

Supplement: Supplementary file 3 [file Image_3.tif]
