# Supplementary material for: Neutrophil autophagy induced by monosodium urate crystals facilitates neutrophil extracellular traps formation and inflammation remission in gouty arthritis
Source: Front Endocrinol (Lausanne). 2023 Sep 22;14:1071630. doi: 10.3389/fendo.2023.1071630 (PMC10557066; doi:10.3389/fendo.2023.1071630)
Supplement: Supplementary file 4 [file DataSheet_1.docx]

**Supplementary Materials and Methods**

**Immunofluorescence**

Isolated neutrophils were cultured on poly-D-lysine coated coverslips followed by 1h incubation for attachment. Subsequently, neutrophils were incubated with MSU crystals for 6h. The supernatant was removed, and the plate was washed gently with PBS. For NETs formation detection, neutrophils were incubated with primary antibodies: anti-H3cit and anti-MPO overnight at 4℃ after fixation with 4% paraformaldehyde and blocking with 5% BSA. Fluorescent antibodies were carried out for 1h at room temperature. Cell nuclei were stained with DAPI. Images were taken using a Zeiss LSM510 microscope (Oberkochen, Germany).

**Immunohistochemistry**

The expression of MPO, ATG7, P53, and PAD4 in the air pouch was detected by immunohistochemistry (IHC). IHC was applied on paraffin-embedded formalin-fixed tissue samples according to standard protocols.

**Western blotting**

The expression of the indicated proteins was assayed using western blotting. The indicated molecules for the analyses were as follows: ATG7, H3cit, p53, and PAD4. The relative levels were normalized against GAPDH in the same samples.

**Enzyme-linked immunosorbent assay**

ELISA (Invitrogen, CA, USA) was applied to measure IL-1β, MCP-1, TNF, and IL-6 levels in the serum of healthy donors and patients. Each experiment was repeated in triplicates.

**Plasmid construction and cell transfection**

Expression vectors containing cDNA encoding p53 were obtained from Sino Biological Inc. (Beijing, China). Plasmids encoding ATG7 were also obtained from Sino Biological Inc. (Beijing, China) and cloned into the Flag vector. Cell transfection was performed with Lipofectamine 3000 (Invitrogen, CA, USA).

**Immunoprecipitation**

Cells were harvested and lysed in immunoprecipitation (IP) buffer (150mM NaCl, 20mM Tris, 1mM EDTA, 1%Triton X-100, pH 7.5 and protease inhibitor). After periodic mixing, the pyrolysis solution was centrifuged for 15min at 12000g. The supernatant was used for concentration determination and immunoprecipitation. Antibodies against Flag (1:100) and rabbit IgG (1:100) were added to the lysates for incubation overnight with GammaBind Plus Sepharose (GE Healthcare, UT, USA). The pellets were washed with cold IP buffer 5 times and detected by western blotting.

**Quantitative real-time PCR**

Total RNA was isolated from neutrophils using TRIzol reagent (Thermo Fisher Scientific, MA, USA). Reverse transcription was carried out using the SuperScript IV Reverse Transcriptase (Thermo Fisher Scientific, MA, USA). Quantitative real-time PCR were performed in Real-Time PCR Detection System (Bio-Rad, CA, USA) with SYBR Green qPCR core reagent. All reactions were run in triplicates. The primers for the analyses were as following: human LC3 I, forward, 5’-GCTACAAGGGTGAGAAGCAGCT-3’, and reverse 5’-CTGGTTCACCAGCAGGAAGAAG-3’; human LC3 II, forward, 5’-GTCCTGGACAAGACCAAGTTCC-3’, and reverse 5’-CCATTCACCAGGAGGAAGAAGG-3’; human ATG3, forward, 5’-ACTGATGCTGGCGGTGAAGATG-3’, and reverse 5’-GTGCTCAACTGTTAAAGGCTGCC-3’; human ATG5, forward, 5’-GCAGATGGACAGTTGCACACAC-3’, and reverse 5’-GAGGTGTTTCCAACATTGGCTCA-3’; human ATG7, forward, 5’-CGTTGCCCACAGCATCATCTTC-3’, and reverse 5’-CACTGAGGTTCACCATCCTTGG-3’; human ATG10, forward, 5’-GGTGATAGTTGGGAATGGAGACC-3’, and reverse 5’-GTCTGTCCATGGGTAGATGCTC-3’; human ATG12, forward, 5’-GGGAAGGACTTACGGATGTCTC-3’, and reverse 5’-AGGAGTGTCTCCCACAGCCTTT-3’;

**RNA sequencing analysis (RNA-seq)**

Total RNA was reversely transcribed into cDNA to generate an indexed Illumina library, and then followed by sequencing at the Beijing Genomics Institute with BGISEQ-500 platform. Differentially expressed genes (DEGs) were defined as >2-fold differential expression vs. the control with an adjusted P value <0.05. DEGs were analyzed by Gene Ontology (AMIGO and DAVID software). The enrichment degrees of DEGs were analyzed with Kyoto Encyclopedia of Genes and Genomes annotations.
